# Reason in All Things

**Published:** 1877-06

**Authors:** 


### *REASON IN ALL THINGS.*

The amount of suffering that is brought upon innocent persons, and particularly upon little children, on account of the extreme views, unreason, and ignorance of those who have them in charge, is simply appalling. If these people were compelled to "practice what they preach," for a little while only, they would be "brought to their senses!" No one questions a true mother's love for her child. She would, if neccessary, give up her own life to save it from suffering and death. It is through mistaken kindness, it is through unpardonable ignorance, that she pursues a course with her child that results in impaired health and an early grave. Infants are daily soused in cold water, tortured with fashionable clothing, habitually starved, sent out with cruel young servants to be broiled in the midday sun, and cuffed and pinched for any manifestation of discomfort. When the summer comes, they are as certain to be stricken down with "Cholera Infantum" as if they had undergone a course of treatment especially designed to bring on that disease. A little "Soothing Syrup" or opium in some other form, will usually complete the work, and put the little sufferers out of their misery forever.

This JOURNAL has labored for nearly a quarter of a century to instruct people how to live in order to enjoy rationally and profitably the gifts of a kind Providence. We have been charged with being the advocates of a meagre diet, such as "Graham bread." "cabbage soup," and other absurdities. We recently received two letters the same day: one from a gentleman in Virginia, who says he has for many years followed the advice of the JOURNAL in the care of his children, "allowing them to eat but little meat, and giving them only a little bread and milk for supper, which they take at five P. M. and nothing after." He fears the "plan of the JOURNAL has not worked well with his children, as they do not seem as strong as he could wish, but he thinks the JOURNAL must be right, for he is acquainted with many people who profess to owe their lives to an observance of its teachings, and he is bound to persevere. We lost no time in writing to our friend, advising him to supply his family with an abundance of wholesome food, and to place no restrictions on its use.

The other letter was from a gentleman residing in Philadelphia, who stated that he was suffering from "Nervous Dyspepsia." He, too, said he was following "the advice of the JOURNAL," and starving himself, eating only crackers and milk, and taking various medicines, but was getting worse instead of better, and he said he was coming to consult with us. He came to New York two weeks ago. We stopped his medicine, and then sent him to Dr. Rae for electrical treatment, and directed that he should have good nutritious food, and regular exercise in the open air. After ten days of this rational treatment, he returned home greatly improved in health and spirits.

The idea we wish to convey is, that we can avoid most of the evils of life by avoiding extremes, and following a rational course, such as is dictated by reason and common sense. A low diet is more destructive to health than habitually over-eating. Thousands of lives are lost for the want of proper medicines, and health is impaired or destroyed by the too free use of improper remedies.

The greatest enemy to health and to long life is habitual constipation. This condition is always alarming, and should be corrected with as little delay as possible, as it always threatens danger from blood poisoning. Regularity of the bowels is essential to health, and where there is regularity we are slow to believe in the existence of any disease unless the symptoms are decidedly prominent. For the permanent cure of constipation we have never employed a remedy equal to "Nelaton's Suppository." Cathartics afford only temporary relief, and increase the difficulty. Injections of all kinds are positively dangerous, if often repeated, on account of the local irritation which may induce the development of piles, fistula, and cancer.

